# Supplementary material for: Proteomic and metabolic profiling reveals APOE4-dependent shifts in whole brain, neuronal, and astrocytic mitochondrial function and glycolysis
Source: bioRxiv. 2025 Jun 16:2025.06.15.659811. Preprint. [Version 1] doi: 10.1101/2025.06.15.659811 (PMC12262718; doi:10.1101/2025.06.15.659811)

bioRxiv preprint doi: https://doi.org/10.1101/2025.06.15.659811; this version posted June 16, 2025. The copyright holder for this preprint (which was not certified by peer review) is the author/funder, who has granted bioRxiv a license to display the preprint in perpetuity. It is made available under aCC-BY 4.0 International license.

Supplemental Figure 1. Whole brain proteomics comparing male and female *APOE* TR mice. A. Number of differentially expressed (DE) proteins for male vs. female mice. B. Volcano plot showing up and down regulated proteins in *APOE3* male vs. female mice. C. Volcano plot showing up and down regulated proteins in *APOE4* male vs. female mice. D. IPA of up and downregulated pathways in *APOE3* male vs. female mice. E. IPA of up and downregulated pathways in *APOE4* male vs. female mice. F. Venn diagram of upregulated protein overlap in *APOE3* and *APOE4* male vs. female mice. G. Venn diagram of down regulated protein overlap in *APOE3* and *APOE4* male vs. female mice.

Supplemental Figure 2. Isolated brain mitochondria proteomics comparing male and female APOE TR mice. A. Volcano plot showing up and down regulated mitochondrial proteins between *APOE3* male and female mice. B. Volcano plot showing up and down regulated proteins between *APOE4* male and female mice. C. IPA showing up and down regulated pathways between *APOE3* male and female mice. D. IPA showing up and down regulated pathways between *APOE4* male and female mice.

Supplemental Figure 3. Stable isotope labeling of additional metabolites in iAstrocytes and iNeurons. iAstrocyte and iNeuron ^{13}C-glucose labeling of A. fructose 1,6-bisphosphate, B. glycerol 3-phosphate, C. lactate, D. alanine, E. NAC. All data are shown as mean ±SD. *p<0.05, **p<0.01.

bioRxiv preprint doi: https://doi.org/10.1101/2025.06.15.659811; this version posted June 16, 2025. The copyright holder for this preprint (which was not certified by peer review) is the author/funder, who has granted bioRxiv a license to display the preprint in perpetuity. It is made available under aCC-BY 4.0 International license.

Supplemental Figure 1 (Whole Brain Proteomics)

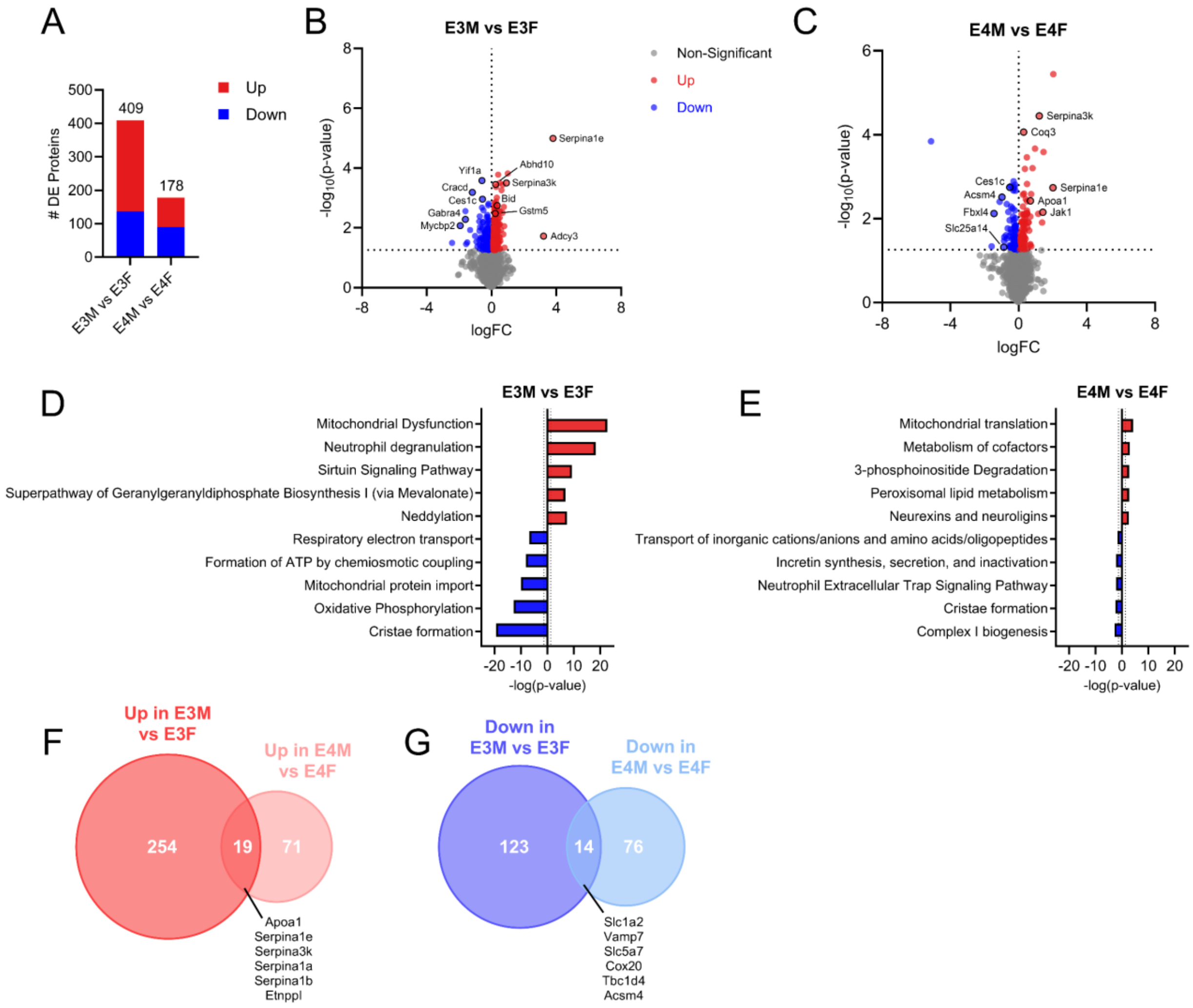

bioRxiv preprint doi: https://doi.org/10.1101/2025.06.15.659811; this version posted June 16, 2025. The copyright holder for this preprint (which was not certified by peer review) is the author/funder, who has granted bioRxiv a license to display the preprint in perpetuity. It is made available under aCC-BY 4.0 International license.

Supplemental Figure 2 (Isolated Brain Mitochondrial Proteomics)

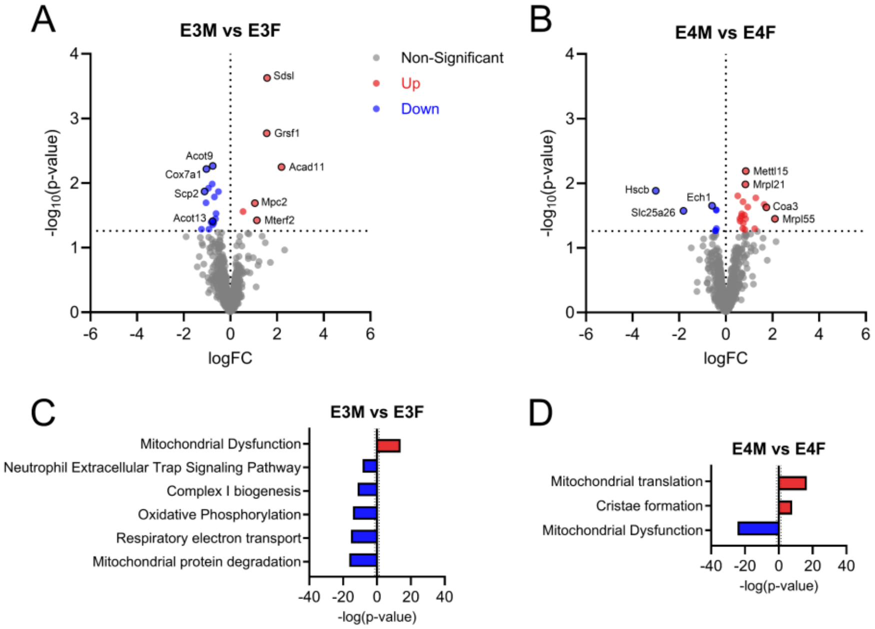

bioRxiv preprint doi: https://doi.org/10.1101/2025.06.15.659811; this version posted June 16, 2025. The copyright holder for this preprint (which was not certified by peer review) is the author/funder, who has granted bioRxiv a license to display the preprint in perpetuity. It is made available under aCC-BY 4.0 International license.

Supplemental Figure 3 (iAstrocyte and iNeuron Metabolomics)

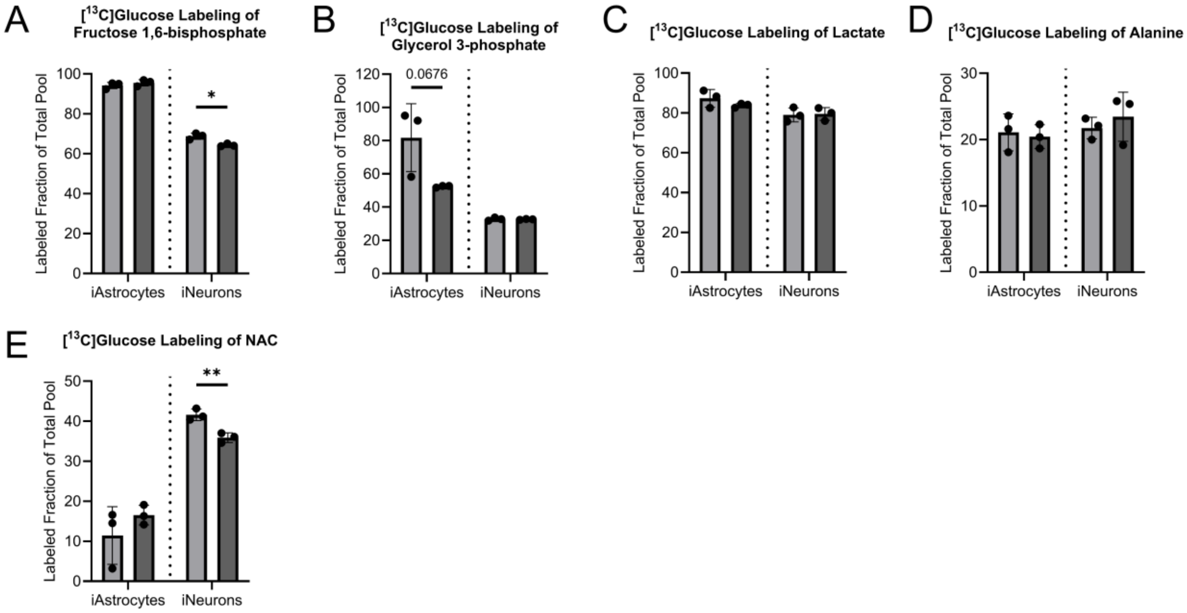

Supplement: 1 [file NIHPP2025.06.15.659811v1-supplement-1.pdf]
